# Supplementary material for: Comparing effects of wearable robot-assisted gait training on functional changes and neuroplasticity: A preliminary study
Source: PLoS One. 2024 Dec 5;19(12):e0315145. doi: 10.1371/journal.pone.0315145 (PMC11620557; doi:10.1371/journal.pone.0315145)
Supplement: S1 File — (PDF) [file pone.0315145.s001.pdf]

Supporting Information

S1 Table. Regions of interest in the functional network

No.	Region	Side	MNI coordinates		
			x	y	z
1	Supplementary motor area	L	-2	-18	73
2	Supplementary motor area	R	4	-4	62
3	Primary motor cortex	L	-6	-42	69
4	Primary motor cortex	R	8	-36	75
5	Cingulate motor area	L	-10	8	36
6	Cingulate motor area	R	4	14	45
7	Dorsal premotor cortex	L	-48	0	51
8	Dorsal premotor cortex	R	52	10	49
9	Ventral premotor cortex	L	-52	2	7
10	Ventral premotor cortex	R	58	4	5
11	Primary somatosensory area	L	-12	-36	62
12	Primary somatosensory area	R	10	-36	71
13	Superior parietal lobule	L	-16	-38	69
14	Superior parietal lobule	R	14	-42	75
15	Inferior parietal cortex	L	-52	-36	18
16	Inferior parietal cortex	R	68	-30	20
17	Secondary somatosensory area	L	-38	-24	14
18	Secondary somatosensory area	R	44	-20	12
19	Primary auditory cortex	L	-40	-28	12
20	Primary auditory cortex	R	48	-20	9
21	Mid-posterior insula	L	-34	-24	16
22	Mid-posterior insula	R	38	-20	14
23	Anterior insula	L	-28	20	9
24	Anterior insula	R	34	26	5
25	Caudate nucleus	L	-10	4	7
26	Caudate nucleus	R	14	4	12
27	Putamen	L	-30	-14	7
28	Putamen	R	30	-4	12
29	Thalamus	L	-16	-18	14
30	Thalamus	R	22	-22	3
31	Cerebellum	L	-16	-38	-21
32	Cerebellum	R	18	-52	-54

L, left side; R, right side

S2 Table. Physical, cognitive, and neuroplastic measures of subject 1

Measures	Pre	Post
**Physical measures**		
Treadmill slope for inclined walking (%)	27.3111	31.4167
Set of squats (n)	9	11
**Cognitive measures**		
Correct answers for cognitive dual task with robot (n)	50	50
Correct answers for cognitive dual task without robot (n)	40	45
**Neuroplastic measures**		
Network efficiency	0.1960	0.2149
Tract integrity of CST	0.6020	0.5834
Tract integrity of CR	0.4759	0.4852
Tract integrity of CC	0.6473	0.6559
Tract integrity of SLF	0.4958	0.4561

CST, corticospinal tract; CR, corona radiata; CC corpus callosum; SLF, superior longitudinal fasciculus.

S3 Table. Physical, cognitive, and neuroplastic measures of subject 2

Measures	Pre	Post
**Physical measures**		
Treadmill slope for inclined walking (%)	28.5833	35.0000
Set of squats (n)	15	12
**Cognitive measures**		
Correct answers for cognitive dual task with robot (n)	32	41
Correct answers for cognitive dual task without robot (n)	49	54
**Neuroplastic measures**		
Network efficiency	0.1915	0.2114
Tract integrity of CST	0.5886	0.6078
Tract integrity of CR	0.4857	0.4841
Tract integrity of CC	0.6577	0.6556
Tract integrity of SLF	0.4560	0.4987

CST, corticospinal tract; CR, corona radiata; CC corpus callosum; SLF, superior longitudinal fasciculus.

S4 Table. Physical, cognitive, and neuroplastic measures of subject 3

Measures	Pre	Post
**Physical measures**		
Treadmill slope for inclined walking (%)	25.3556	31.5833
Set of squats (n)	13	14
**Cognitive measures**		
Correct answers for cognitive dual task with robot (n)	51	62
Correct answers for cognitive dual task without robot (n)	61	74
**Neuroplastic measures**		
Network efficiency	0.1334	0.1277
Tract integrity of CST	0.5922	0.6317
Tract integrity of CR	0.4910	0.5090
Tract integrity of CC	0.6672	0.6854
Tract integrity of SLF	0.4799	0.4812

CST, corticospinal tract; CR, corona radiata; CC corpus callosum; SLF, superior longitudinal fasciculus.

S5 Table. Physical, cognitive, and neuroplastic measures of subject 4

Measures	Pre	Post
**Physical measures**		
Treadmill slope for inclined walking (%)	29.1056	32.6722
Set of squats (n)	10	17
**Cognitive measures**		
Correct answers for cognitive dual task with robot (n)	40	51
Correct answers for cognitive dual task without robot (n)	45	50
**Neuroplastic measures**		
Network efficiency	0.1665	0.1417
Tract integrity of CST	0.6114	0.5776
Tract integrity of CR	0.4863	0.4936
Tract integrity of CC	0.6628	0.6638
Tract integrity of SLF	0.4638	0.4834

CST, corticospinal tract; CR, corona radiata; CC corpus callosum; SLF, superior longitudinal fasciculus.

S6 Table. Physical, cognitive, and neuroplastic measures of subject 5

Measures	Pre	Post
**Physical measures**		
Treadmill slope for inclined walking (%)	29.5388	31.4500
Set of squats (n)	9	12
**Cognitive measures**		
Correct answers for cognitive dual task with robot (n)	51	56
Correct answers for cognitive dual task without robot (n)	46	52
**Neuroplastic measures**		
Network efficiency	0.1322	0.1642
Tract integrity of CST	0.5998	0.5128
Tract integrity of CR	0.4668	0.4346
Tract integrity of CC	0.6530	0.6006
Tract integrity of SLF	0.4449	0.4113

CST, corticospinal tract; CR, corona radiata; CC corpus callosum; SLF, superior longitudinal fasciculus.

S7 Table. Physical, cognitive, and neuroplastic measures of subject 6

Measures	Pre	Post
**Physical measures**		
Treadmill slope for inclined walking (%)	35.0000	40.0000
Set of squats (n)	12	12
**Cognitive measures**		
Correct answers for cognitive dual task with robot (n)	39	37
Correct answers for cognitive dual task without robot (n)	38	39
**Neuroplastic measures**		
Network efficiency	0.2104	0.1882
Tract integrity of CST	0.5507	0.6061
Tract integrity of CR	0.4517	0.4772
Tract integrity of CC	0.6273	0.6615
Tract integrity of SLF	0.4229	0.4601

CST, corticospinal tract; CR, corona radiata; CC corpus callosum; SLF, superior longitudinal fasciculus.

S8 Table. Physical, cognitive, and neuroplastic measures of subject 7

Measures	Pre	Post
**Physical measures**		
Treadmill slope for inclined walking (%)	25.2833	30.3667
Set of squats (n)	13	13
**Cognitive measures**		
Correct answers for cognitive dual task with robot (n)	36	58
Correct answers for cognitive dual task without robot (n)	43	63
**Neuroplastic measures**		
Network efficiency	0.2089	0.2177
Tract integrity of CST	0.5885	0.5744
Tract integrity of CR	0.4620	0.4515
Tract integrity of CC	0.6374	0.6277
Tract integrity of SLF	0.4323	0.4233

CST, corticospinal tract; CR, corona radiata; CC corpus callosum; SLF, superior longitudinal fasciculus.

S9 Table. Physical, cognitive, and neuroplastic measures of subject 8

Measures	Pre	Post
**Physical measures**		
Treadmill slope for inclined walking (%)	35.0000	40.0000
Set of squats (n)	13	15
**Cognitive measures**		
Correct answers for cognitive dual task with robot (n)	69	71
Correct answers for cognitive dual task without robot (n)	56	68
**Neuroplastic measures**		
Network efficiency	0.2015	0.2391
Tract integrity of CST	0.5251	0.5494
Tract integrity of CR	0.4371	0.4515
Tract integrity of CC	0.6224	0.6372
Tract integrity of SLF	0.4237	0.4347

CST, corticospinal tract; CR, corona radiata; CC corpus callosum; SLF, superior longitudinal fasciculus.

S10 Table. Physical, cognitive, and neuroplastic measures of subject 9

Measures	Pre	Post
**Physical measures**		
Treadmill slope for inclined walking (%)	33.4889	40.0000
Set of squats (n)	13	15
**Cognitive measures**		
Correct answers for cognitive dual task with robot (n)	36	41
Correct answers for cognitive dual task without robot (n)	32	41
**Neuroplastic measures**		
Network efficiency	0.1669	0.2280
Tract integrity of CST	0.5481	0.5554
Tract integrity of CR	0.4806	0.4771
Tract integrity of CC	0.6458	0.6441
Tract integrity of SLF	0.4516	0.4516

CST, corticospinal tract; CR, corona radiata; CC corpus callosum; SLF, superior longitudinal fasciculus.

S11 Table. Physical, cognitive, and neuroplastic measures of subject 10

Measures	Pre	Post
**Physical measures**		
Treadmill slope for inclined walking (%)	30.5330	35.0000
Set of squats (n)	13	14
**Cognitive measures**		
Correct answers for cognitive dual task with robot (n)	41	40
Correct answers for cognitive dual task without robot (n)	47	52
**Neuroplastic measures**		
Network efficiency	0.2474	0.2682
Tract integrity of CST	0.5774	0.5854
Tract integrity of CR	0.4576	0.4581
Tract integrity of CC	0.6326	0.6308
Tract integrity of SLF	0.4366	0.4381

CST, corticospinal tract; CR, corona radiata; CC corpus callosum; SLF, superior longitudinal fasciculus.

S12 Table. Physical, cognitive, and neuroplastic measures of subject 11

Measures	Pre	Post
**Physical measures**		
Treadmill slope for inclined walking (%)	29.2778	35.0000
Set of squats (n)	11	13
**Cognitive measures**		
Correct answers for cognitive dual task with robot (n)	39	45
Correct answers for cognitive dual task without robot (n)	40	39
**Neuroplastic measures**		
Network efficiency	0.2645	0.2485
Tract integrity of CST	0.5984	0.5968
Tract integrity of CR	0.5000	0.4903
Tract integrity of CC	0.6712	0.6660
Tract integrity of SLF	0.5006	0.4949

CST, corticospinal tract; CR, corona radiata; CC corpus callosum; SLF, superior longitudinal fasciculus.

S13 Table. Physical, cognitive, and neuroplastic measures of subject 12

Measures	Pre	Post
**Physical measures**	`	
Treadmill slope for inclined walking (%)	35.0000	30.1389
Set of squats (n)	14	16
**Cognitive measures**		
Correct answers for cognitive dual task with robot (n)	61	56
Correct answers for cognitive dual task without robot (n)	56	61
**Neuroplastic measures**		
Network efficiency	0.2127	0.2695
Tract integrity of CST	0.6026	0.6121
Tract integrity of CR	0.4904	0.4954
Tract integrity of CC	0.6734	0.6829
Tract integrity of SLF	0.4657	0.4736

CST, corticospinal tract; CR, corona radiata; CC corpus callosum; SLF, superior longitudinal fasciculus.
