# Supplementary material for: Comparing effects of wearable robot-assisted gait training on functional changes and neuroplasticity: A preliminary study
Source: PLoS One. 2024 Dec 5;19(12):e0315145. doi: 10.1371/journal.pone.0315145 (PMC11620557; doi:10.1371/journal.pone.0315145)
Supplement: Attachment — Submitted filename: Response to Reviewers.docx [file pone.0315145.s002.docx]

**Responses to Reviewers**

We would like to thank the editor and reviewers for their constructive comments. We have addressed their specific concerns below and have made the suggested changes to the manuscript. Point-by-point responses to the editor’s and reviewers’ comments are presented in blue font as follows.

Editor

1. Please ensure that your manuscript meets PLOS ONE's style requirements.

- We revised the manuscript style to meet PLOS ONE’s style requirement.

2. We note that the grant information you provided in the ‘Funding Information’ and ‘Financial Disclosure’ sections do not match. When you resubmit, please ensure that you provide the correct grant numbers for the awards you received for your study in the ‘Funding Information’ section

- The Funding Information is correct. There was no space to update the Financial Disclosure to match the Funding Information during this revised manuscript submission. Therefore, the following disclosure has been added to the cover letter as requested.

*“This study was supported by T-ROBOTICS (G01210547) and the National Research Foundation of Korea (NRF) grant funded by the Korean government (MSIT). (No. RS-2023-00208884).”*

3. We note that your Data Availability Statement is currently as follows: All relevant data are within the manuscript and its Supporting Information files. Please confirm at this time whether or not your submission contains all raw data required to replicate the results of your study.

- In this study, measures of physical function, cognitive function, and neuroplasticity at pre- and post-training time points were used to calculate the effect size of the proposed RAGT. All raw data on physical function, cognitive function, and neuroplasticity for each participant before and after the intervention have been included in the supporting information file (S1 File). The reference to the S1 File can be found in the revised manuscript.

Reviewer #1: In this paper, the technique of Robot-Assisted Gait Training (RAGT) is established to show improvement in gait training for patients with gait disorders. The technique is insightful and offers interesting outcomes. However, there are certain points which I would like the authors to consider amending in this document as follows:

1. The abstract does not explicitly link the findings with the actual objective of the study, set out in the beginning of the abstract. Please revise the latter sections of the abstract.

- In the abstract, objectives of the preliminary study are to verify the feasibility of the proposed RAGT design and to assess and compare the effect sizes of various measurement variables, including physical, cognitive, and neuroimaging by the RAGT. We revised the latter sections (results and conclusions parts) of the abstract as follows.

*“All participants underwent 10 sessions of RAGT without any adverse incidents, and the feasibility of the proposed RAGT design, consisting of preferred speed walking, fast speed walking, inclined walking, and squats, was validated. Variables related to physical and cognitive abilities significantly improved, but those related to neuroplasticity did not. The effect size of physical ability was “very large,” whereas that of cognitive ability was “medium-to-large.” The effect sizes of functional and structural neuroplasticity showed “medium” and “very small,” respectively. The effect size of the RAGT varied depending on the measured variables, with the effect size being the greatest for physical ability, followed by cognitive ability, functional neuroplasticity, and structural neuroplasticity. The proposed RAGT design affects cognitive and neuroplastic effects beyond the physical effect directly affected by RAGT. This study highlights that while RAGT can positively influence cognitive outcomes beyond physical benefits, more intensive interventions may be required to elicit significant neuroplastic changes. This preliminary study offers useful information for researchers interested in designing robot-assisted training by investigating the potential extent of neuroplastic effects.“*

2. In the introduction section, the authors need to ground the study in the previous literature, which has not been done.

- We added the previous literatures and revised the manuscript in the Introduction section as follows.

*“Gait function involves multiple regions and networks of the central nervous system, as well as the musculoskeletal system. Accordingly, RAGT is anticipated to influence not only gait performance but also cognitive function and neuroplasticity. Several studies reported that RAGT improved cognitive function, such as dual-task performance and cognitive assessment score, in stroke patients [1-3]. Also, previous neuroimaging studies reported that RAGT modulated brain activation and power in the specific frequency band in the motor-specific areas and facilitated white matter integrity in stroke patients [4-6]. Previous studies have separately examined the effects of RAGT on cognitive function, functional neuroplasticity, and structural neuroplasticity, respectively. To the best of our knowledge, studies that simultaneously investigate cognitive function, functional neuroplasticity, and structural neuroplasticity within a single study are limited.”*

3. Moreover, the introduction section should elaborate about the RAGT technique with evidence and justification.

- We elaborated about the RAGT technique with evidence and justification in the Introduction section as follows.

*“Recently, robot-assisted gait training (RAGT), which uses advanced electromechanical devices to support body weight and automate the gait process, has been used to enhance the gait function of people whose mobility has been affected by aging and neurological disorders [7-11]. RAGT can provide highly intensive and repetitive training with consistent and precise movements, which can be difficult to achieve with conventional therapy [12]. Because these characteristics of RAGT are crucial for motor learning and neuroplasticity, RAGT is particularly beneficial for individuals with aging and neurological impairment. According to previous clinical studies, RAGT improved gait function, including gait speed, cadence, stride length, step width, single support time, and cardiopulmonary metabolic efficiency in elderly adults [7]. Also, in RAGT studies for patients with neurological disorders, gait speed, endurance, balance, and functional ambulation were improved in stroke, Parkinson’s disease, and spinal cord injury patients, and abnormal gait patterns and muscle strength were better in children with cerebral palsy [8-11].”*

4. The methodology section lacks addressal to the exact model and type of wearable RAGT device and training were utilized in the experiment. Moreover, there is no pictorial evidence and no citation-based evidence to justify the use of the particular Myosuit.

- We revised descriptions about the exact model and type of wearable RAGT device utilized in the training in the Materials and Methods section as follows.
- Fig 1 was also updated to show the Myosuit being worn.

*“The Myosuit, a fully untethered and autonomous soft, cable-driven exosuit weighing 5.5 kg, is designed to assist across daily life activities and to enable intensive training for strength, endurance, and balance activities with minimal interference with the user’s movements [13]. The exosuit in use is shown in Fig 1. Therefore, the Myosuit is suitable as this study device for exploring the training effects of high-intensity lower limb exercises in daily activities for healthy individuals. The device consists of a backpack-style motor unit, a textile vest with a waist belt, and polymer knee orthoses. Adjustable polymer springs assist passive hip flexion, while ultra-high-molecular-weight polyethylene cables, driven by electric motors in the backpack, provide active assistance. The cables are routed across the user's lower body, coupling hip and knee extension in an underactuated manner. The Myosuit can automatically detect gait phases and assist the knee and hip extensor muscles using one cable routed across the hip and knee joints and passive elastic bands. During walking, cable tension is applied during the stance phase to assist hip and knee extension. The assistance level can be customized for each leg, providing a high level of personalization to meet the user’s training requirements [13].”*

5. In the process of RAGT, there needs to be justification of why 150% of the preferred walking speed was kept.

- In this study, during the fast speed walking, the speed was set at 150 % of the preferred walking speed. According to previous studies [14-16], fast walking speeds range between 120-150 % of the preferred walking speed. In this study, as it involves relatively high-intensive training, the speed was set at 150 %. We added the information in the revised manuscript.

6. Citations for how previous similar trainings were done are needed in the methodology section.

- We added the descriptions and references related to training design in the Materials and Methods section as follows.

*“RAGT generally involves gait training either on a treadmill or overground at the preferred waking speed or fast walking speed. Additionally, activities such as up-hill and down-hill walking, stairs ascending and descending, and squatting can be selected based on the participant’s functional status or training goal [17, 18]. In this study, RAGT involved the preferred speed walking, fast speed walking, inclined walking on a treadmill, and squatting on the ground for 40 minutes. Thus, the training was composed of a combination of conventional exercises. The unique aspect lies in the personalization of treadmill incline during inclined walking, adjusted according to the individual’s physical capacity and training effects.”*

7. The method for gauging accuracy of answers in the tasks is unclear.

- We revised the method for gauging the number of correct answers in the dual task in the Materials and Methods and Results sections as follows.

*“The participants were asked to perform a phonemic verbal fluency task while walking on a treadmill. The participants in the phonemic verbal fluency task were required to listen to the consonant presented by the experimenter and generate as many words as possible that started with the given consonant within a 15 second timeframe (two consonants per dual task walking block) [19].* *The number of correct and non-repetitive words generated by the participant that begin with the given consonant during the dual task timeframe is counted and recorded by the experimenter.”*

*“The improvement in cognitive ability during gait was examined through changes in the number of correct answers during the cognitive dual task before and after 10 training sessions. The number of correct answers refers to the count of accurate and non-repetitive words generated by the participant during the verbal fluency task while walking on a treadmill.”*

8. Further explanation is needed for explaining medium to large effect size in the findings section.

- We changed a sentence for explaining medium-to-large effect size in the Results section as follows.

*From:*

*The changes in correct answers during the cognitive dual task showed a medium-to-large effect size (with robot, d=0.71; without robot, d=1.25).*

*To:*

*“In the changes in correct answers during the cognitive dual task with robot, a medium effect size was observed (d=0.71), while a large effect size was observed in the dual task without the robot (d=1.25), indicating an overall medium-to-large effect size.”*

9. A control and experimental group were needed to measure the efficacy of the RAGT.

- We know that a control group is needed to measure the efficacy of the RAGT. However, we needed a preliminary study like this one before conducting a randomized controlled trial. The purpose of this study was to confirm the feasibility of the experiment, including whether participants could tolerate 40 minutes of training and the durability of the device, as well as to explore the effect size for calculating the sample size and designing an experimental-control study with the device and protocol. Furthermore, as evidenced by our use of MRI and fNIRS in this study, we are particularly interested in the neuroplastic effects of RAGT. To the best of our knowledge, previous studies have not simultaneously investigated the physical, cognitive, structural, and functional neuroplastic effects of RAGT. Therefore, this study not only serves as a necessary precursor for designing experiments to verify our hypothesis but also provides useful information for researchers interested in the neuroplastic effects of RAGT and those designing similar experiments. Therefore, we believe that this study can provide additional value to the readers in the RAGT field. The opinions are covered throughout the revised manuscript.

10. Statistical analysis and verification are needed to gauge the ‘positive effect of RAGT on brain plasticity’.

- We performed a statistical analysis on neuroplastic changes. As presented in the results, unlike the changes in physical and cognitive function, neuroplastic changes did not reach statistical significance. The measure with the greatest change was the efficiency of the gait-related network, which represented functional neuroplastic change and showed marginal significance (p=0.0872). Other functional and structural neuroplastic measures demonstrated only a trend of positive change after training. Therefore, we revised the term "positive effect of RAGT on brain plasticity" in the discussion section to "positive trend of RAGT" to more accurately reflect the findings, and we toned down the discussion on neuroplastic changes accordingly. Although statistical significance was not observed in neuroplastic changes, we believe that this study can provide insights into the effect size of neuroplastic changes compared to physical and cognitive function changes when RAGT is applied to the same participants. Additionally, it can offer valuable information for estimating the sample size needed to observe functional neuroplastic changes in future studies.

11. The findings section is very sketchy and needs to include vital comparisons and analysis to establish significance of the outcome.

- We revised Table 1 and added sentences in the Results section to convey the results more clearly by comparing among physical, cognitive, and neuroplastic measures as follows.

From*:*

Table 1. Changes in white matter integrity of major nerve tracts to investigate structural neuroplasticity

| White matter tracts | *Pre-training* | *Post-training* | *t* | *p* |
| --- | --- | --- | --- | --- |
| Corticospinal tract | 0.5821 (0.0267) | 0.5827 (0.0323) | 0.06 | 0.9503 |
| Corona radiata | 0.4738 (0.0188) | 0.4756 (0.0221) | 0.43 | 0.6790 |
| Corpus callosum | 0.6498 (0.0173) | 0.6510 (0.0243) | 0.19 | 0.8554 |
| Superior longitudinal fasciculus | 0.4562 (0.0261) | 0.4589 (0.0283) | 0.39 | 0.7013 |

Data are expressed as the mean (standard deviation).

To:

Table 1. Changes in physical, cognitive, and neuroplastic measures

| Measures | Pre | Post | *p* | Cohen’s d | ES |
| --- | --- | --- | --- | --- | --- |
| **Physical measures** | |  |  |  |  |
| Treadmill slope for inclined walking (%) | 30.29 (3.58) | 34.39 (3.79) | <0.001 | 1.32 | Very large |
| Set of squats (n) | 12.08 (1.93) | 13.67 (1.83) | 0.0372 | 0.68 | Medium |
| **Cognitive measures** | |  |  |  |  |
| Correct answers for cognitive dual task with robot (n) | 45.42 (11.16) | 50.67 (10.33) | 0.0311 | 0.71 | Medium |
| Correct answers for cognitive dual task without robot (n) | 46.08 (8.40) | 53.17 (11.45) | 0.0012 | 1.25 | Large |
| **Neuroplastic measures** | |  |  |  |  |
| Network efficiency | 0.1943 (0.0401) | 0.2099 (0.0464) | 0.0872 | 0.54 | Medium |
| Tract integrity of CST | 0.5821 (0.0267) | 0.5827 (0.0323) | 0.9503 | 0.02 | Very small |
| Tract integrity of CR | 0.4738 (0.0188) | 0.4756 (0.0221) | 0.6790 | 0.12 | Very small |
| Tract integrity of CC | 0.6498 (0.0173) | 0.6510 (0.0243) | 0.8554 | 0.05 | Very small |
| Tract integrity of SLF | 0.4562 (0.0261) | 0.4589 (0.0283) | 0.7013 | 0.11 | Very small |

Data are expressed as the mean (standard deviation). ES, effect size; CST, corticospinal tract; CR, corona radiata; CC corpus callosum; SLF, superior longitudinal fasciculus.

*“The changes in physical, cognitive, and neuroplastic measures are summarized in Table 1. The effect size for physical ability was categorized as "very large," while cognitive ability demonstrated a "medium-to-large" effect size. The effect sizes for functional and structural neuroplasticity were "medium" and "very small," respectively. The effect of RAGT varied across the different measures, showing the largest effect size for physical ability, followed by cognitive ability, functional neuroplasticity, and structural neuroplasticity.”*

12. The manuscript lacks a section on conclusion, which is very important to comprehensively summarize the whole study.

- We revised a conclusion paragraph in the Discussion section by summarizing study results as follows.

*“**This preliminary study confirmed the feasibility of implementing a high-intensity RAGT training protocol utilizing the device, as all participants successfully completed the proposed training program. Additionally, we assessed the effect sizes induced by the RAGT from various perspectives, including physical function, cognitive function, and neuroplasticity, by measuring physical, cognitive, functional and structural neuroimaging measures before and after the training. The effect sizes were observed in the following order: physical function, cognitive function, and neuroplastic changes. RAGT demonstrated significant effects on both physical and cognitive functions in this study. From a neuroplasticity perspective, while a positive trend was noted, the effect was relatively weak. This finding suggests that more intensive interventions and personalized strategies in terms of intervention design may be necessary to induce neuroplastic changes beyond the improvements observed in physical and cognitive functions. Furthermore, the measurement methods for physical, cognitive, and neuroplastic changes, as well as the quantitative information regarding the effect sizes of neuroplastic changes compared to those of physical and cognitive functions, may assist in designing future studies, including intervention, examination, and sample size calculation, for researchers interested in the neuroplastic effects induced by RAGT.”*

Reviewer #2: The manuscript presents the feasibility of the robot assisted gait-training (RAGT)-based study design to evaluate the physical, cognitive, and neuroimaging effect. 10 healthy adults participated in this study. Walking (preferred sped, fast, and squats) with and without RAGT on treadmill with and without inclined surface is practiced to measure physical strength, magnetic resonance imaging (MRI) scanner and a functional near-infrared spectroscopy (fNIRS) device is used to measure and evaluate cognitive and neuroimaging (cognitive and neuroplasticity) behavior. The manuscript is well written and well structured.

Following are the suggestions to improve the quality of manuscript.

1. The sample size is very small to estimate the results or impact of the study. Authors mentioned total of 12 healthy adults out of which 10 are male adults but no information is presented regarding the rest of two participants.

- The objectives of the preliminary study are to verify the feasibility of the proposed RAGT design and to assess and compare the effect sizes of various measurement variables, including physical, cognitive, and neuroimaging by the RAGT. Therefore, the sample size is small. However, we believe this study can offer useful information for researchers interested in designing robot-assisted training by providing the effect size of neuroplastic change compared to physical and cognitive function and investigating the potential extent of neuroplastic effects.
- We revised the sentence related to the participants’ information as follows.

*“In total, 12 healthy adults (10 males, 2 females; mean age: 25.5 ± 3.7 years) were enrolled in this study.”*

2. The discussion section only includes few studies to verify and compare results. All the studies verified the current study results but this is very limited in scope of this study. Although this is mentioned in the limitations, however this limited the novelty and contribution of this study. If all the conclusions are already explored in the literature then what this study implies?

- In this study, the soft, cable-driven exosuit was used for the RAGT, and the RAGT was composed of a combination of the preferred speed walking, fast speed walking, inclined walking, and squatting. Also, personalized training was adopted by adjusting the incline according to the individual’s physical capacity and training effects. The distinction of this study lies in its examination of the effects of the RAGT from various perspectives, including physical, cognitive, and neuroplasticity outcomes, using different devices, training protocols, and measures compared to previous studies. Nevertheless, we think the individual findings on physical function, cognitive function, and neuroplasticity can be in line with the reports of previous individual studies.
- Previous studies have primarily reported on the effects of RAGT on physical function, while some studies have also investigated its effects on cognitive function or neuroplasticity. However, to the best of our knowledge, studies that simultaneously investigate cognitive function, functional neuroplasticity, and structural neuroplasticity within a single study are limited. This study provides information for comparing the effect size of RAGT from the perspectives of physical function, cognitive function, and both functional and structural neuroplasticity. The effect sizes were observed in the following order: physical function, cognitive function, and neuroplastic changes. RAGT demonstrated significant effects on both physical and cognitive functions in this study. From a neuroplasticity perspective, while a positive trend was noted, the effect was relatively weak. This finding suggests that more intensive interventions and personalized strategies in terms of intervention design may be necessary to induce neuroplastic changes beyond the improvements observed in physical and cognitive functions. Furthermore, the measurement methods for physical, cognitive, and neuroplastic changes, as well as the quantitative information regarding the effect sizes of neuroplastic changes compared to those of physical and cognitive functions, may assist in designing future studies, including intervention, examination, and sample size calculation, for researchers interested in the neuroplastic effects induced by RAGT.
- To more clearly convey the results and significance of this study, we revised the Introduction and Discussion sections by adding relevant literatures and modifying the descriptions. Additionally, Table 1 in the Results section were modified in the revised manuscript.

3. In cognitive-related activity, why all the channels are placed at prefrontal cortex, since the participant is performing motor activity, premotor cortex should be included it will provide additional insights of combined mental workload and cognitive-related activity both in prefrontal and premotor cortex.

- We agree with the reviewer's comment. We wanted to acquire signals throughout the entire frontal cortical regions, including the prefrontal and premotor cortex, during the dual task, but our fNIRS device does not allow for channel scalability or flexible channel placement. In future research, we plan to establish an experimental setup that allows for measurements across the entire frontal cortex to gain additional insights into the combined mental workload and cognitive-related activity.

4. Does Fig-4 represents average cognitive activity of all trials and participants or single-trial of example participant? This should be mentioned in the caption or description.

- This figure shows the average activation of the prefrontal cortex during cognitive dual task obtained from fNIRS data of all trials and participants. We revised the figure caption in the revised manuscript.

5. The pixel quality of fig-3 is not good and it is difficult to analyze the figure. Please improve the quality of this figure.

- We remade figure 3 in the revised manuscript to improve pixel quality.

6. There are many mistakes in references formatting, please review thoroughly.

- We reviewed the reference format and revised formatting in the revised manuscript.

7. The contribution of the study with respect to neuroplastic effect are very limited. The authors discussed the limitation in the discussion section “Although no statistically significant results or relatively small effect sizes were observed in our study” mainly because of small sample size. This decrease the impact, novelty and contribution of this study as compared to the claims of this study.

- Our response to the reviewer's second comment may partially address the concern in this comment. As a preliminary study, this research aims to provide useful information for designing future studies, as no prior data exist regarding the Myosuit and specific training used. Regarding the neuroplastic effect, although no statistically significant changes were observed in this study design, power analysis using statistical values and effect sizes could help determine the appropriate sample size for future research. Upon internal review, it appears that a sample size of at least twice the current number of participants, i.e., more than 20 individuals, is necessary to adequately assess the functional neuroplastic effects using the efficiency of the gait-related network of RAGT in this study. Also, the smaller effect size of functional neuroplasticity compared to those of physical and cognitive functions suggests that more intensive and personalized intervention strategies may be required to induce neuroplastic changes beyond the observed improvements in physical and cognitive functions.
- We are well aware of the limitations of this study, as mentioned in the manuscript. However, we believe that our study can provide valuable insights into experimental design and measurement methods for researchers interested in RAGT. We have made significant revisions to convey the contribution of this study effectively in the revised manuscript.

**References**

1.	Akinci M, Burak M, Kasal FZ, Özaslan EA, Huri M, Kurtaran ZA. The Effects of Combined Virtual Reality Exercises and Robot Assisted Gait Training on Cognitive Functions, Daily Living Activities, and Quality of Life in High Functioning Individuals With Subacute Stroke. Perceptual and Motor Skills. 2024;131(3):756-69.

2.	Kayabinar B, Alemdaroğlu-Gürbüz İ, Yilmaz Ö. The effects of virtual reality augmented robot-assisted gait training on dual-task performance and functional measures in chronic stroke: a randomized controlled single-blind trial. Eur J Phys Rehabil Med. 2021;57(2):227-37.

3.	Kim Y, Park C, Yoon B, You JH. Bolstering Cognitive and Locomotor Function in Post-Stroke Dementia Using Human–Robotic Interactive Gait Training. Journal of Clinical Medicine. 2023;12(17):5661.

4.	Kim DH, Kang CS, Kyeong S. Robot-assisted gait training promotes brain reorganization after stroke: A randomized controlled pilot study. NeuroRehabilitation. 2020;46(4):483-9.

5.	Kim H, Park G, Shin J, You J. Neuroplastic effects of end-effector robotic gait training for hemiparetic stroke: a randomised controlled trial. Sci Rep 2020; 10: 12461. PUBMED.

6.	Shin J, An H, Yang S, Park C, Lee Y, You SJH. Comparative effects of passive and active mode robot-assisted gait training on brain and muscular activities in sub-acute and chronic stroke 1. NeuroRehabilitation. 2022;51(1):51-63.

7.	Lee H-J, Lee S, Chang WH, Seo K, Shim Y, Choi B-O, et al. A wearable hip assist robot can improve gait function and cardiopulmonary metabolic efficiency in elderly adults. IEEE transactions on neural systems and rehabilitation engineering. 2017;25(9):1549-57.

8.	Nam KY, Kim HJ, Kwon BS, Park J-W, Lee HJ, Yoo A. Robot-assisted gait training (Lokomat) improves walking function and activity in people with spinal cord injury: a systematic review. Journal of neuroengineering and rehabilitation. 2017;14:1-13.

9.	Park Y-H, Lee D-H, Lee J-H. A Comprehensive Review: Robot-Assisted Treatments for Gait Rehabilitation in Stroke Patients. Medicina. 2024;60(4):620.

10.	van Hedel HJ, Meyer-Heim A. Robot-Assisted Gait Training for Children and Youth with Cerebral Palsy. Cerebral Palsy. 2020:2797-816.

11.	Xue X, Yang X, Deng Z. Efficacy of rehabilitation robot-assisted gait training on lower extremity dyskinesia in patients with Parkinson's disease: A systematic review and meta-analysis. Ageing Research Reviews. 2023;85:101837.

12.	Sivan M, O'Connor RJ, Makower S, Levesley M, Bhakta B. Systematic review of outcome measures used in the evaluation of robot-assisted upper limb exercise in stroke. Journal of rehabilitation medicine. 2011;43(3):181-9.

13.	Schmidt K, Duarte JE, Grimmer M, Sancho-Puchades A, Wei H, Easthope CS, et al. The myosuit: Bi-articular anti-gravity exosuit that reduces hip extensor activity in sitting transfers. Frontiers in neurorobotics. 2017;11:57.

14.	Fukuchi CA, Fukuchi RK, Duarte M. Effects of walking speed on gait biomechanics in healthy participants: a systematic review and meta-analysis. Systematic reviews. 2019;8:1-11.

15.	Laufer Y. Effect of age on characteristics of forward and backward gait at preferred and accelerated walking speed. The Journals of Gerontology Series A: Biological Sciences and Medical Sciences. 2005;60(5):627-32.

16.	Ridge ST, Henley J, Manal K, Miller F, Richards JG. Biomechanical analysis of gait termination in 11–17 year old youth at preferred and fast walking speeds. Human Movement Science. 2016;49:178-85.

17.	Haufe FL, Schmidt K, Duarte JE, Wolf P, Riener R, Xiloyannis M. Activity-based training with the Myosuit: a safety and feasibility study across diverse gait disorders. Journal of neuroengineering and rehabilitation. 2020;17:1-11.

18.	Basla C, Mariani G, Wolf P, Riener R, van Hedel HJ. Enhancing walking efficiency of adolescents with neurological impairments using an exosuit for ambulatory activities of daily living. Frontiers in Robotics and AI. 2024;11:1335733.

19.	Dorfman M, Herman T, Brozgol M, Shema S, Weiss A, Hausdorff JM, et al. Dual-task training on a treadmill to improve gait and cognitive function in elderly idiopathic fallers. Journal of Neurologic Physical Therapy. 2014;38(4):246-53.
